# Management of encephalomyocarditis virus infection in Italian pig farms: a case report

**DOI:** 10.1186/s12917-023-03611-6

**Published:** 2023-02-24

**Authors:** A. Scollo, C. Mazzoni, A. Luppi

**Affiliations:** 1 Department of Veterinary Sciences, University of Torino, Grugliasco, 10095 Torino, Italy; 2 Swivet Research sas, 42123 Reggio Emilia, Italy; 3 Istituto Zooproflattico Sperimentale della Lombardia e dell’Emilia-Romagna, 43126 Parma, Italy

**Keywords:** Encephalomyocarditis virus, Pigs, Acetylsalicylic acid, Support therapy

## Abstract

**Background:**

Encephalomyocarditis virus (EMCV) has been isolated from many animals, frequently as the cause of fatal myocarditis, but pigs are the most susceptible domestic specie. The virus was isolated in swine farms since 1958 in Panama and Europe from cases of sudden death in young pigs, and the main origin of outbreaks has been assumed to be local rodent populations. There is no treatment for the disease.

**Case presentation:**

The clinical case describes an outbreak of encephalomyocarditis virus in a farrowing (farm A) and a weaning (farm B) site, with mortality that reached 24.2% in suckling piglets and 7.7% in weaners. The farms were located in an endemic Italian area, and the outbreak was characterised by high mortality with sudden death and clinical signs due to heart failure (trembling, dispnea and fever). The rodents control program was the key action in managing the outbreak. However, in the weaning site, the lack of rodent program in some unexplored areas of the barn (false ceiling) was responsible of a longer time of resolution of the outbreak. An unusual support treatment approach from human medicine suggestion was also applied using acetylsalicylic acid for its antiphlogistic and antithrombotic effects.

**Conclusions:**

To control the rodent population in a pig farm is often difficult and requires a deep knowledge also of the rodents habits. Considering the lack of treatment for the disease and the absence of available vaccines in several Countries, acetylsalicylic acid might be of interest for further studies as an important support for pigs’ recovery.

## Background

Encephalomyocarditis virus (EMCV) is a member of the family *Picornaviridae*, genus *Cardiovirus*. It was first isolated from cases of acute fatal myocarditis in a chimpanzee in Florida [1] and encephalomyelitis in a rhesus monkey in Uganda [2]. Since then, EMCV has been isolated from many animals, frequently as the cause of fatal myocarditis. Acute fatal myocarditis in piglets caused by EMCV was first described in Panama in 1958 [3], hereafter pigs have been considered to be the most susceptible domestic specie. In the mid-1980s, EMCV infection was also associated with reproductive failure in sows [4, 5], depending on the strain. In Europe, EMCV was first reported to be the cause of disease in pigs in 1986, in both Italy and Greece [6, 7]. After 1986, the virus was usually isolated in Italy from cases of sudden death in young pigs, with a total of 72 outbreaks during 2000 and 2001, recorded in the endemic area of southern Lombardy or the bordering regions of Veneto and Emilia Romagna. The disease mostly caused low mortality, occurring as sporadic episodes of sudden death of a few (< 10) suckling or weaned piglets. However, in some outbreaks the disease was more severe, resulting in 100–400 deaths [8]. In the Italian endemic area, a seroprevalence between 5.7 and 14.5% was reported, with peaks higher than 60% in farms with clinical encephalomyocarditis [8].

Currently two main mechanisms of transmission are considered for EMCV in domestic pigs: 1) infection of pigs that ingest either infected faeces or urine or the carcasses of infected rodents, often through contaminated feed or water; or 2) pig-to-pig transmission, even if pigs can excrete the virus only for a short period. Transplacental transmission may also occur [9–11]. The main origin of outbreaks has been assumed to be local rodent populations [12]. In rats experimentally infected with a myocardial EMCV strain, no clinical or macroscopic lesions were observed in any organs, and virus was isolated from Peyer’s patches and thymus from 3 days until 60 days post-inoculation. This finding indicated that this tissue represented a site of persistence after oral infection [13].

The course of the infection in swine is influenced by the virulence of the strain, exposure dose, individual susceptibility and immune status of the animals. Thirty hours after experimental infection some animals can die with typical lesions in the heart and tonsils and 3 days post-infection the virus can be isolated also from blood. The highest virus titres are recovered from heart muscle, and myocardial lesions are predominant at necropsy as a sign of being the target organ for the virus replication. Myocarditis caused by EMCV seem to be a consequence of pro-inflammatory cytokines (IL-1β, tumor necrosis factor-α, IL-6) production [14].

Clinical disease is developed more often in younger pigs, especially in the first weeks of life, and is commonly characterized by an acute manifestation with sudden death due to myocardial failure. Other clinical signs, such as anorexia, listlessness, trembling, staggering, paralysis, or dyspnoea have also been observed, sometimes with temperatures up to 41 °C. The disease is often restricted to one barn or some pens, and deaths are more frequent in the late afternoon, when pigs are more active. Before dying, dyspnea and squealing can be noticed. The chronic myocarditis can occur after an occasional recovery or infections in pigs from post-weaning age to adulthood, with subclinical disease and occasional mortality [15]. Pigs dying from the acute phase may show only epicardial haemorrhages and no other gross lesions, except for hydropericardium, hydrothorax and pulmonary oedema. The most characteristic lesions, more frequently observed in growing pigs than in suckling piglets, are found in the myocardium where multiple foci of necrosis are observed. Histopathology shows characteristic necrosis with or without mineralization in the heart, where a positive immunohistochemical reaction localized to the cytoplasm of myocardial cells is observed. Meningitis, mononuclear cells perivascular infiltration and neural degeneration may be observed in the brain [15].

Conclusive diagnosis of EMCV might be obtained by the histopathological lesions (non-suppurative interstitial myocarditis or encephalitis), virus isolation in culture, and RT-PCR for a more sensitive and specific methods of diagnosis. Serological tests for the detection of serum antibodies against EMCV, such as Virus neutralization (VN) and ELISA, are also used. Neutralizing antibodies can be detected 5–7 days post-infection and may persist from 6 months to 1 year, while maternal immunity can persist for at least 2 months [15].

There is no treatment for the disease, but avoiding stress or excitement of the pigs at risk in the acute phase may minimize mortality. However, in human medicine, clinical management of myocarditis is more developed towards a therapeutic intervention with the aim to reduce phlogosis and persistent cardiac dysfunction in people survived to an acute episode. Non-steroidal anti-inflammatory drugs, in particular acetylsalicylic acid, are a cornerstone of treatment for acute cardiac disease in human due to their antiphlogistic and antithrombotic effects [16]. Aim of the present clinical report was to describe an outbreak of EMCV in both a farrowing (farm A) and a weaning (farm B) site in Italy, where a support treatment approach from human medicine suggestion was also applied.

## Case presentation

The outbreak described in the present clinical case occurred in a conventional intensive farrowing unit (farm A) and in a weaning site (farm B) of a multisite herd located in the province of Brescia, in Northern Italy (Lombardy). A total number of 1200 sows Large White x Landrace breed were allocated in the farm A, organized with a 3-weeks batch system and weaning age of piglets of 28 days. Both farm A and farm B had a rigid external biosecurity protocol and good hygiene level, with a rodents control program applied. The piglets were systematically vaccinated intramuscularly against *Mycoplasma hyopneumoniae* and porcine circovirus type 2 on day 21 of age. They were then vaccinated against Aujeszky’s disease virus as dictated by the national protocol (three times during life; the first on day 70 of age, the second 21 days later; the third at 160 days of age; both the first and the second vaccination injected in the weaning site). Sows were vaccinated for atrophic rhinitis (at 70 and 90 days of gestation), parvovirus combined with *Erysipelothrix rhusiopathiae* every 4 months, and Aujeszky’s disease virus at 70, 92 and 160 days of age. All the piglets underwent to surgical castration and iron injection at 3 days of life. Piglets belonging to each batch in farm A were moved every 3 weeks to a different weaning unit, and specifically every 6 weeks in the farm B, located 5 km far from the farm A. The farm B had a single barn divided in two sections separate by a door, with 6 identical rooms per each section and 8 identical pens within each room. The barn had a plastic slatted floor (according to EU regulations), forced ventilation, water available through nipple drinkers and ad libitum dry meal feeding. There were nearly 1800 piglets in each batch, allotted in one of the two sections of the barn (a total of two batches in the barn, the first 6 weeks older than the second), where the system in place was All-in/All-out. Animals arrived in the farm B at 7 kg of weight, and were moved to the fattening site when they were 30 kg weigh (11 weeks after weaning). Stocking density was kept within the legal EU limits (until 30 kg of body weight: 0,30 m2/head; EU Directive 2008/120/CE). Other general information of case farms A and B are reported in Table 1.

**Table 1:** General information of case farms A and B

	Farm A	Farm B
Type of farm	Farrowing unit	Weaning unit
Herd size and management	1200 sows, three-weeks batches	1800 pigs/batch, two batches present in two different sectors of the barn, the first 6 weeks older than the second
Drinking water	Ground water	Ground water
Cleaning and disinfection	Dirt was manually removed; crates were then soaked with a commercial product, removed using water under high pressure (220 bar) and a disinfection product contained chloride was applied. Crates were left empty for 2–3 days.	Cold water was used for cleaning the pens. Pens were first soaked with a commercial product. Dirt was removed by using water under high pressure (220 bar). The applied disinfection product contained chloride. The stand-empty period was 1 week.
Stressful operations	Cross-fostering of piglets from one sow to another; surgical castration and iron injection (day 3 of life); vaccination toward *M. hyopneumoniae* and porcine circovirus type 2, skin tattoo for Parma ham regulation (day 21 of life).	Moving little piglets from a pen to another for size balancing intra-batches; vaccination towards Aujeszky’s disease (day 70 of life).
Rodent control program	A professional company was responsible for the rodent control. No excessive trails such as feces, damage to insulation and corpses of rat and mice were observed.	A professional company was responsible for the rodent control. Trails, such as damage to insulation and presence of rat feces were visible.

The outbreak started in the mid-September in farm A and in the early October in farm B, and was characterized by sudden death and high mortality in piglets of different ages. Batch that experienced the outbreak in farm B did not show any related problem during its lactation period in farm A. In the farrowing unit (farm A), the mortality rate ranged from 0 to 100% in different litters (total mortality 24.2%, vs 14.2% of the previous 6 batches), with occasional anorexia, trembling, dispnea and fever (41 °C) in older suckling piglets (from 21 to 28 days of age); in the weaning site (farm B) the mortality rate increased from 0.74% (average of the last 4 weaning cycles) to 4.85%, with more frequent clinical symptoms often accompanied to squealing before dying.

**Fig. 1:**
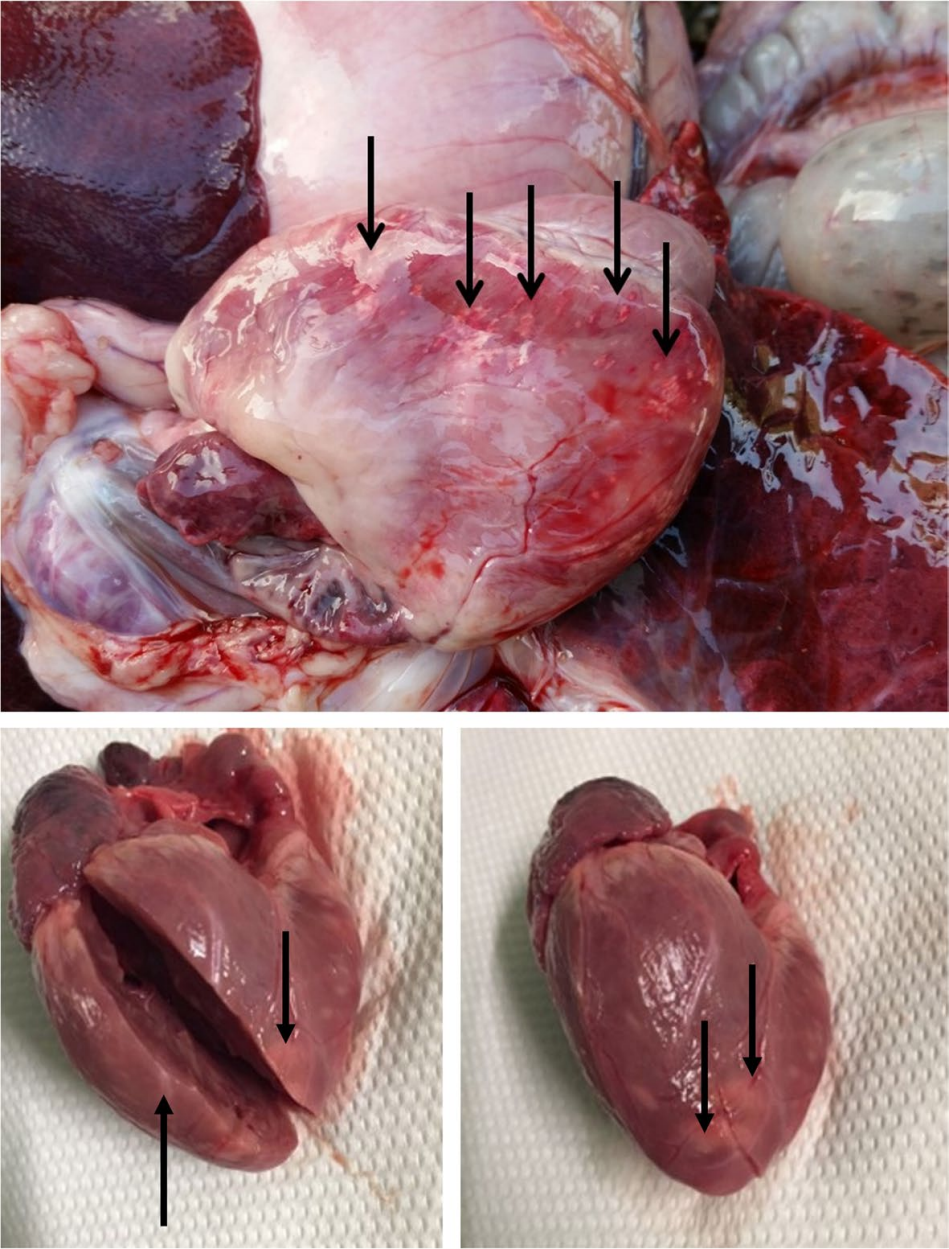
Heart of a weaned piglet. White foci of necrosis can be observed in the myocardium (arrows)

Nine piglets (5 from the farm A and 4 from the farm B) were collected and sent to the laboratories of *Istituto Zooprofilattico Sperimentale della Lombardia e dell’Emilia Romagna* for necropsies and analysis. The main gross lesions observed during the necropsies were myocardial foci of necrosis (Fig. 1), hydrothorax, meningeal congestion, hydropericardium and pulmonary congestion and oedema.

Differential diagnosis considered: 1) Foot and mouth disease (FMD; gross and microscopic cardiac lesions may be indistinguishable from EMCV); 2) Parvovirus infection (PPV; can induce a non-suppurative myocarditis); 3) Vitamin E/Se deficiency (Mulberry Heart Disease); 4) Edema disease (*Escherichia coli*); 5) Streptococcosis (*Streptococcus suis*); 6) Porcine reproductive and respiratory syndrome (PRRSV); 7) Aujeszky’s disease (PRV); 8) Classical swine fever (CSF).

At necropsies, samples of several tissues were collected and tested with appropriate diagnostic methods to confirm or exclude the differential diagnosis considered above. Bacteriological investigations, RT-PCRs for PRRSV and for EMCV and a PCR for PRV were performed following standardized methods (Table 2).

**Table 2:** Laboratory investigations performed on samples collected during the necropsies on 9 piglets

Samples	Methods	Etiologic agent/aim	Results
Brain, spleen; kidney	Bacteriology (brain was cultured on blood agar supplemented with NAD and Gassner agar as well as spleen and kidney were cultured on blood agar and Gassner agar)	*Streptococcus suis, Haemophilus parasuis, Escherichia coli* and other possible bacterial agents	Negative
Spleen, lung	RT-PCR	PRRSV	Negative
Brain	PCR	PRV	Negative
Spleen, lymph nodes	ELISA (antigen)	CSF	Negative
Heart	RT-PCR	EMCV	Positive
Brain, lung, heart, thymus, lymph nodes, liver, stomach, intestine, kidney, spleen, pancreas, bone marrow	Histology and immunohistochemistry (EMCV mAb 3E5)	- microscopic evaluation of tissues - confirmation of the results obtained with the other diagnostic methods	Immunohistochemistry detected EMCV in cardiac muscle cells, Purkinje fibres, endothelial cells of blood vessels and macrophages (Fig. 3). The heart showed myocarditis characterized by an interstitial lymphoplasmacytic infiltration (Fig. 2)
Heart	Cell culture (African green monkey kidney epithelial cell – MARC 145)	- isolation of EMCV	Isolated (Fig. 4)
Cell culture with cytopathic effect	ELISA (antigen)	- EMCV identification	Identified

**Fig. 2:**
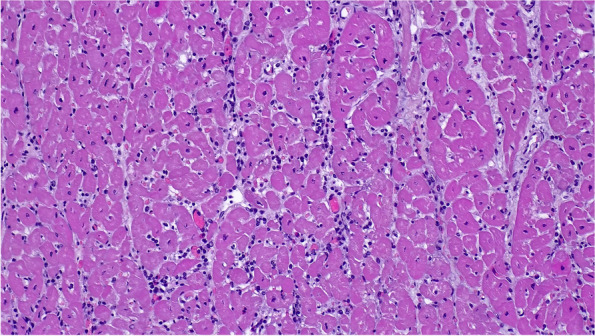
Histopathology of a pig’s heart. Myocarditis is characterized by an interstitial lymphoplasmacytic infiltration (Hematoxylin-eosin, 40× magnification)

**Fig. 3:**
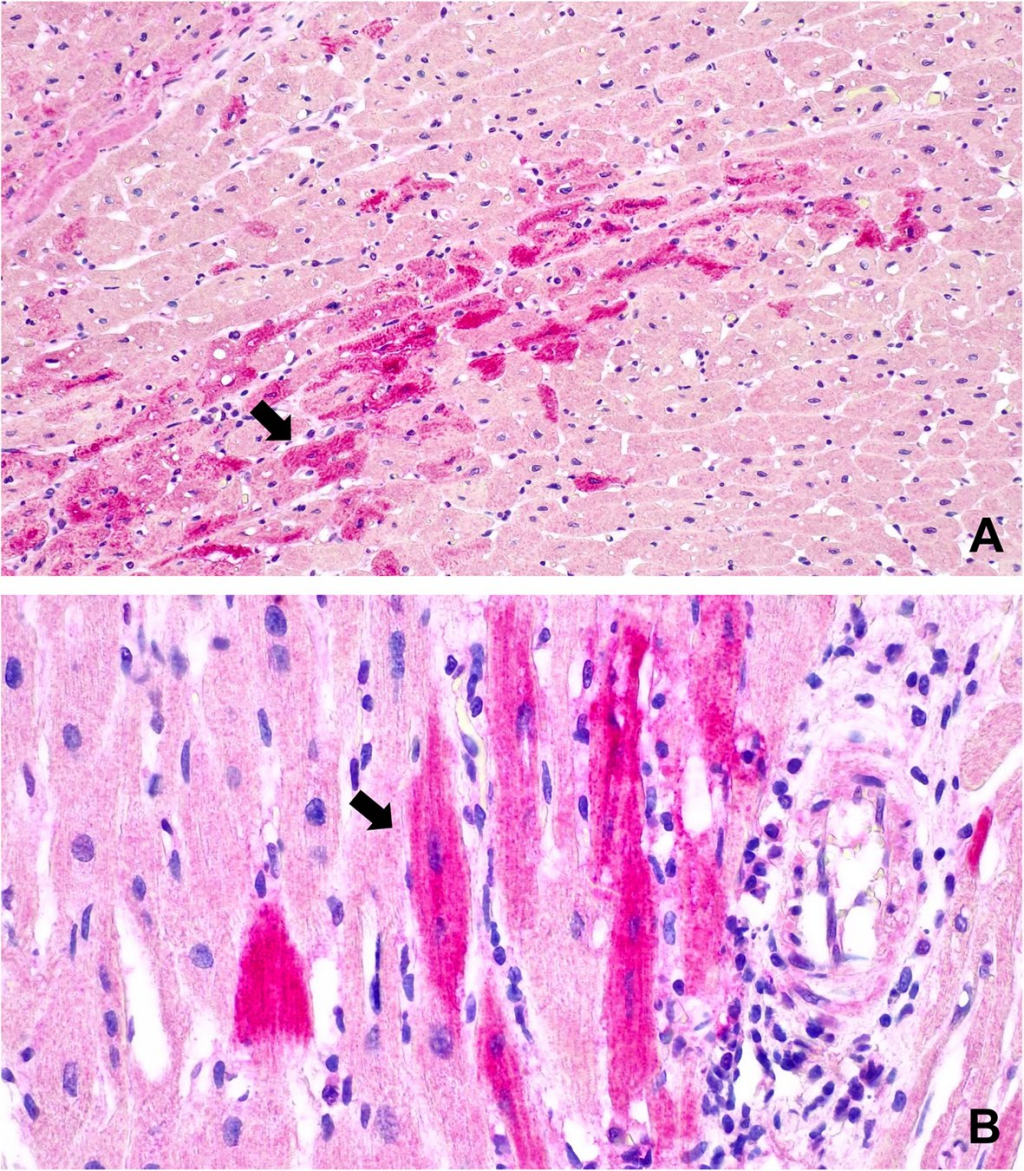
Heart of pig affected by Encephalomyocarditis. Cardiomyocytes are immunoreactive for EMCV (arrows), immunostained with mAb 3E5. **A** 20X; **B** 60X

**Fig. 4:**
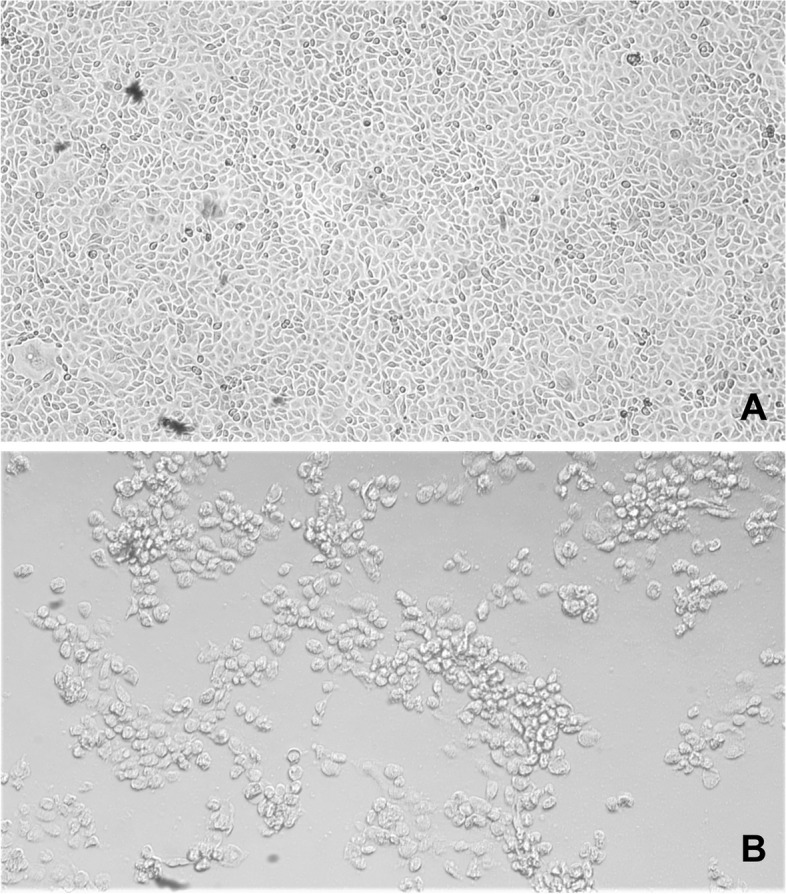
MARC 145 Cell culture. **A** Negative control: uninfected cell monolayer (10× magnification); **B** Evident cytopathic effect due to EMCV, 72 hours after the infection (20× magnification)

The bacteriological investigations performed did not show bacterial pathogens. The RT-PCR for PRRSV, the PCR for PRV and the direct ELISA for CSF gave negative results for all samples tested. All the samples tested for EMCV by RT-PCR resulted positive. The histopathology of the heart showed myocarditis characterized by an interstitial lymphoplasmacytic infiltration (Fig. 2). Using immunohistochemistry (IHC), EMCV was detected in cardiac muscle cells, Purkinje fibres, endothelial cells of blood vessels and macrophages (Fig. 3). The results of RT-PCRs, PCR, histopathology, IHC and cell cultures (Fig. 4) allowed an aetiological diagnosis of encephalomyocarditis due to EMCV in both farm A and farm B.

Considering the lack of treatment for the disease and the absence of available vaccines in Italy, the main interventions were 1) minimize stress or excitement of the piglets (e.g. limiting moving piglets from one sow to another in farm A, and from different pens in farm B); 2) improvement and intensification of the rodent programs in both farms. Moreover, an implementation of the hygiene of the farms was applied using iodine-based preparation, as a segregation of feed in dedicated closed areas when possible.

After the outbreak in farm A, lasted 4 weeks (two batches were involved), no other cases were shown. Differently, mortality in farm B continued to increase also in the sequent cycles, arising to a peak of 7.7% the month later the first outbreak. In the analysis of the rodents’ programs performed in farm B, the lack of action on the false ceiling of the barn emerged. A second and more intensive rodent control program was then performed, including a deep removal of all the abandoned old material in the false ceilings, and a new placement of baiting-points was made in the area under the roof.

Concurrently with the second action towards rodents in the weaning site, a treatment with 4.2 g/100 kg body weight q 24 h of acetylsalicylic acid was administered for 5 days. Thereafter, using a therapeutic approach adopted in human suffering of myocarditis and survived to myocardial infarct, a low-dose acetylsalicylic acid (1.2 g/100 kg body weight q 24 h) was administered for 2 weeks.

Death decreased and stopped 4 days after rodents control program and the administration of the low-dose acetylsalicylic acid. A similar treatment of low-dose acetylsalicylic acid was repeated in the same animals before the time of vaccination, as a stressing event for the animals, without any other clinical evidence of disease or mortality. After implementing a more stringent and frequent rodent control no EMCV outbreak has occurred in farm A and farm B until the time of submitting this paper.

## Discussion and conclusions

The clinical case describes an outbreak of EMCV in a farrowing and a weaning site, with mortality that reached 24.2% in suckling piglets and 7.7% in weaners. Clinical signs developed during the outbreak were typical of the disease, with sudden death in younger piglets and more evident trembling, dyspnea and staggering before dying in older animals. EMCV is more pathogenic to the myocardium of younger animals and this can explain the higher death rate in lactating piglets, and the absence of reproductive problems in the farrowing unit confirms findings previously reported for Italy in a European survey conducted by Maurice et al. [8], that highlighted Italy suffering from the myocardial form of EMCV only. The farms are located in the endemic small area of southern Lombardy where several outbreaks occurred between 1997 and 2001, even if the disease mostly caused low mortality, occurring as sporadic episodes of sudden death of a few suckling or weaned piglets. The duration of the outbreak corresponds to other reported in the survey, as well as the seasonality (autumn-winter period), probably due to rodents migration for food shortages or changes in rodent population density as already described [8]. The severity of the outbreak of this clinical case, differently to other cases with low losses reported in Italy and in various countries, might be explained by differences in the pathogenicity of the EMCV strains [17], the available infectious dose [18], and/or the susceptibility of the pigs, for example by age and breed. Although some studies [19, 20] have demonstrated antigenic stability in EMCV, strain differences in biological characteristics are known. Also differences in pathogenicity within the same or between different isolates [17, 21] and differences in tissue tropism are indicated between various strains [10].

The failure of the first rodents control program in farm B highlights the importance of knowledge of rodents’ activity. A correct rodents’ program is a necessary measure to prevent pigs from becoming infected with diseases transmitted by rodents. There are farm structures that support rodents’ settlement and these sites are preferred by rodents searching for shelter. In the present clinical case, the presence of stacks of construction materials and at heaps of old materials in the false ceiling of the barn was responsible of the failure of a proper rodents’ control program. Endepol and Klemann [22] reported that rodent-infested farms had almost four times the number of sites with abandoned old materials than farms without rats. Removing such structures and accessing hidden areas such as the false ceiling would obviously reduce the hazard of rodents, but often this benefit is still underestimated.

In the present clinical case, an unusual therapeutic approach was applied using acetylsalicylic acid as used in human medicine to reduce persistent cardiac dysfunction in people survived to an acute episode of myocardial disease. Non-steroidal anti-inflammatory drugs, acetylsalicylic acid in particular, are recommended in all clinical conditions linked with myocardial disease due to their antithrombotic/antiphlogistic prevention. Considering that myocarditis caused by EMCV infection seem to be a consequence of pro-inflammatory cytokines (IL-1β, tumor necrosis factor-α, IL-6) production after virus replication in the heart [14, 23], acetylsalicylic acid was administered at full dose for its antiphlogistic properties [24] at the beginning of the therapy.

After the first 5 days of full dose treatment, given the potential of aspirin to cause dose-dependent impairment of gastric cytoprotection and endothelial thromboresistance, literature encourages to use in humans the lowest dose of aspirin shown to be effective in maintenance of antithrombotic properties. The available evidence supports the use of daily doses of aspirin in the range of 75 to 100 mg for the long-term prevention of serious vascular events in high-risk patients, such as 4 times lower than full dose therapy [25]. In the present clinical case, a low-dose acetylsalicylic acid treatment (4 times lower than full dose) was administered for 2 weeks. Antithrombotic properties of acetylsalicylic acid acts primarily by interfering with the biosynthesis of cyclic prostanoids: TXA2, prostacyclin, and other prostaglandins. It irreversibly inhibits COX-1 by acetylation of serine-530 and induces a long-lasting functional defect in the platelets. The resultant decrease in production of prostaglandins and TXA2 probably accounts for much of the antithrombotic effect [26].

Limits of the approach to the clinical case were 1) absence of a serological survey in sows in order to better understand serological status of the herd and consequently understand the potential presence of variable protective maternal immunity within different litters, in the attempt to explain why mortality ranges from 0 to 100%; 2) absence of serological investigation in piglets, in order to understand if immune status of animals could have influenced the severity of the disease and to explore seroprevalence in the herd; 3) absence of experimental analysis (control group) on effects of acetylsalicylic acid treatment on pigs suffering of myocarditis. For the last point, further studies are suggested to investigate the effect of acetylsalicylic acid treatment by an experimental point of view.

## Data Availability

Not applicable.

## Abbreviations

EMCV: Encephalomyocarditis virus
VN: Virus neutralization
FMD: Foot and mouth disease
PPC: Parvovirus infection
PRRSV: Porcine reproductive and respiratory syndrome
PRV: Aujeszky’s disease
CSF: Classical swine fever
IHC: Immunohistochemistry
